# Health-Related Quality of Life of Former Lead Workers in Brazil

**DOI:** 10.3390/ijerph121114084

**Published:** 2015-11-03

**Authors:** Martha Carvalho Pereira Teixeira, Fernando Martins Carvalho, Liliane Lins

**Affiliations:** Post-Graduate Program in Health, Environment, and Work, School of Medicine, Federal University of Bahia, Largo do Terreiro de Jesus, s/n. Centro Histórico, Salvador, Bahia 40026-010, Brazil; E-Mails: marthateixeira6@yahoo.com.br (M.C.P.T.); liliane.lins@ufba.br (L.L.)

**Keywords:** quality of life, retirement, occupational health, metallurgy, lead poisoning

## Abstract

Little is known about the health-related quality of life of former lead workers. Using the Short-Form 36 Questionnaire (SF-36), a cross-section design study evaluated the health-related quality of life of 186 former workers of a lead smelter that operated in Santo Amaro da Purificação, Brazil, from 1960 to 1993, when it closed down. The smelter had very poor occupational and environmental hygiene standards. The health-related quality of life of former lead workers was low, compared to population-based and other nosological groups from Brazil. Former lead workers who indicated metal poisoning, difficulty getting another job and who could not get another job after dismissal by the smelter presented poorer health-related quality of life. Former lead workers with poor health-related quality of life form part of the huge occupational liability left by the Santo Amaro lead smelter.

## 1. Introduction

Exposure to lead has been associated with multiple health effects and damages to most physiological human systems. However, studies about long-term effects of occupational lead exposure are still scarce. Thus, little is known about the late effects of lead exposure on the physical and mental health of former lead workers. However, among occupationally exposed populations, the effects of lead can be seen in gastrointestinal symptomatology, anemia, cardiovascular diseases, hearing loss, nervous system (nerve conductive effects, on behavior, and cognition), the reproductive system, genotoxicity, and carcinogenicity [1]. Among lead workers, blood lead levels above 1.93 µmol/L (40 µg/dL) have been associated with increased risk of high blood pressure, impaired renal function, and cancer. Even low blood lead levels in adults have been associated with an increased risk of cardiovascular disease and all-cause mortality. In adults, blood lead levels above 0.48 µmol/L (10 µg/dL) have been associated with cancer, cardiovascular and all-cause mortality [2].

Studies measuring lead peak concentration in tibia have associated cognitive decline with past lead exposure among organolead manufacturing workers [3] and among the elderly chronically exposed to environmental lead [4]. Blood lead levels were associated with frailty (diminished physiological reserve in multiple systems with a decreased ability to cope with acute stressors) among older people from the USA [5].

Studies into the effects of lead upon human health usually adopt the toxicological approach, identifying and measuring objective hazards/disruptions caused by the metal on specific organs and systems. This approach implicitly assumes, but does not demonstrate, that the observed effects would affect the individual health-related quality of life. Quality of life has been defined as “*individuals’ perception of their position in life in the context of the culture and value systems in which they live and their relation to their goals, expectations, standards and concerns*” [6]. Health-related quality of life is difficult to define but may be thought of as those aspects of self-perceived well-being that are related to or affected by the presence of disease, treatment and health policies [7]. Considering that health-related quality of life is an essentially subjective concept, measurement can only be properly performed from the point of view of the affected individual. 

The Short-Form 36 Questionnaire (SF-36) provides a generic, subjective measure of functional health and well-being from the patient's point of view. It has been extensively used for monitoring specific populations with a wide range of diseases and health conditions such as arthritis, cancer, cardiovascular disease, chronic obstructive pulmonary disease, depression, gastro-intestinal disease, migraine headaches, hypertension, kidney disease, lower back pain, musculoskeletal conditions, neuromuscular conditions, osteoarthritis, psychiatric diagnoses, rheumatoid arthritis, sleep disorders, stroke, surgical procedures, transplantation and trauma [8]. 

To the best of our knowledge, no study has measured the health-related quality of life of lead smelter former workers. A primary lead smelter, originally controlled by the multinational group Peñarroya and subsequently by a Brazilian group, operated in Santo Amaro da Purificação, Brazil, from 1960 to 1993, producing approximately 22,000 tons/years of lead ingots. The smelter had very poor occupational and environmental hygiene standards. 

In 1974, 57 workers, randomly selected from smelter production areas, presented very high mean levels of PbB (93.62 ± 33.27 µg/100 mL) and urinary ALA (18.68 ± 10.25) [9]. Renal dysfunction, defined as a serum creatinin level ≥ 1.5 mg/dl, was present in 32.7% of the 57 workers from the production area and in 2.3% of workers from a control population [10]. In December 1991, the Regional Labor Authority [11] has analyzed 40 air samples from the smelter; half of them showed lead concentrations above 100 μg/m^3^.

Over 33 years, the smelter produced some 500,000 tons of industrial solid residues (dross, slag) containing 2%–3% of lead. About 20% to 30% out of this total was given to the local Prefecture that paved city streets with the smelter dross. Many cases of paraoccupational lead and cadmium poisonings were subsequently reported in fishermen [12] and children [13,14] from Santo Amaro.

According to the 2010 Census, Santo Amaro da Purificação had 57,800 inhabitants. The Human Development Index (HDI) was 0.646, ranking below the national median (0.727), occupying the 3186th position among the 5565 Brazilian municipalities. Life expectancy was 72 years; mean monthly family per capita income was US$ 185 (or 333 Brazilian reais; 1 US$ = 1.80 real) and only 49% of people aged 18 years or more had complete fundamental education (nine school years) [15]. 

In this work we evaluated the health-related quality of life of former workers of a lead smelter that operated in Santo Amaro da Purificação, State of Bahia, Brazil, from 1960 to 1993.

## 2. Methods

We conducted an exploratory, descriptive study. The local Lead, Cadmium, and Mercury Victims’ Association kept a databank with 456 former lead workers from Santo Amaro City. A sample of 304 (66.7%) individuals was randomly selected from this databank, but 114 were excluded for several reasons (address not found, former worker had moved away, or severe disease that prevented the worker from answering the questionnaire). Four formal refusals were received. The final sample comprised 186 (54.4%) out of the 342 eligible former workers.

A structured questionnaire was applied to the former lead workers to obtain information on sociodemographic characteristics, occupational history and health-related quality of life. Interviews were performed by an economist (MCPT) and four students of medicine, from May to July 2008, in the Lead, Cadmium, and Mercury Victims’ Association headquarters. The Victims’ Association called the associates to attend to an interview. If a former lead worker did not attend the interview when invited, visitsto his/her home address were made to carry out the there. If the second visit was unsuccessful, the individual was discarded from the sample. 

The Brazilian Portuguese [16] validated version of the SF-36 Health Survey version 1, 4-week recall form, as recommended by QualityMetric Incorporated, was used in this study. The eight scales—physical functioning (PF), role limitations due to physical problems (RP), bodily pain (BP), general health perceptions (GH), vitality (VT), social functioning (SF), role limitations due to emotional problems (RE), and mental health (MH)—using the original 0 to 100 algorithms (raw scores) and the two summary components (Physical Component Summary and Mental Component Summary) were scored by QualityMetric Health Outcomes^TM^ Scoring Software 4.0 [17]. Subsequently, the raw scores were normalized, based on a mean of 50 and a standard deviation of 10, taking the US general population as reference. Normalized scores allow us to compare scales and component summaries, because of their comparable variance [17]. QualityMetric Health OutcomesTM licensed this study with the number QM025904. The Statistical Package for the Social Sciences [18] set of programs was used for data processing and calculating t-tests for independent samples. 

The study was approved by the Ethical Review Board from Maternidade Climério de Oliveira, Federal University of Bahia, protocol number 236/2008, respecting Brazilian Health National Council Resolution 466/12, as well as the Helsinki Declaration, 2008. All workers were informed and signed the two copies of the consent form approved by the Ethical Board. A copy of the form stayed with the worker. 

## 3. Results

### 3.1. Demographic, Socioeconomic and Occupational Characteristics of the 186 Former Lead Workers

There were 180 males and six females; mean age of 56.2 ± 10.4, varying from 36 to 86 years; 65.1% had less than eight schooling years; 68.3% were married, 79.6% were head of their families, and 66.1% were Black. One hundred and seventynine (96.2%) out of the 186 workers had formal and stable employment contracts with the lead smelter; three had short term contracts, and four had worked for outsourced enterprises. At the time of the interview, only 7.5% of the former lead workers had a formal job contract; 36.6% had informal jobs; 18.2% were unemployed, and 37.6% were retired. Sixty-six per cent of the former lead workers had monthly incomes below US$ 231 (or 415 reais, the current Brazilian minimal wage); and 67.8% of them lived in families with total monthly family income below US$ 461.

Mean working time in the lead smelter was 7.1 ± 6.3 years. Thirty per cent out of the 186 former lead workers were on duty on the day the smelter has closed down.

### 3.2. The Health-Related Quality of Life of Former Lead Workers

All SF-36 domains scores were very low. GH, PH, RF, BP were particularly low; these domains have major contributions to the construction of the Physical Component Summary measure that was accordingly very low (32.2 ± 8.6). RE (role limitations due to emotional problems) was the most affected domain (33.7 ± 12.4), among those (VT, SF, MH and RE) that most contribute to the mental component summary of the health-related quality of life of these former lead workers (Table 1).

**Table 1:** SF-36 raw and normalized scores (mean ± SD) of 186 former lead workers from Santo Amaro da Purificação lead smelter, Brazil, 2008.

SF-36 Domain/Component Summary	Rawn Score	Normalized Score
Physical Functioning (PF)	37.5 ± 25.0	30.9 ± 10.5
Role Physical (RP)	18.7 ± 28.7	33.2 ± 8.1
Bodily Pain (BP)	38.8 ± 21.7	36.6 ± 9.3
General Health (GH)	27.4 ± 20.5	30.0 ± 9.6
Vitality (VT)	40.0 ± 22.6	42.0 ± 10.7
Social Functioning (SF)	56.9 ± 28.8	38.4 ± 12.5
Role Emotional (RE)	31.4 ± 39.2	33.7 ± 12.4
Mental Health (MH)	55.3 ± 21.1	38.7 ± 12.6
Physical Component Summary (PCS)	---	32.2 ± 8.6
Mental Component Summary (MCS)	---	40.5 ± 12.0

Former workers from the Santo Amaro lead smelter presented lower mean SF-36 scores than other Brazilian populations. Comparison groups included randomly selected population-based samples of healthy individuals aged > 60 years [19] and individuals with stroke sequelae [19]; individuals retired because of disability [20], and groups of patients with severe diseases such as chronic renal failure undergoing hemodialysis [21]; post-polio syndrome [22]; femoral fractures [23,24,25]; and chronic obstructive pulmonary disease at long-term oxygen therapy [26]. Only the group of patients with chronic obstructive pulmonary disease, at long-term oxygen therapy, showed SF-36 scores as low as those of Santo Amaro former lead workers (Table 2). 

SF-36 mean scores of former lead workers did not differ markedly after stratification by demographic and socioeconomic characteristics (age, racial group, schooling, being the head of the family, number of persons in the family, and monthly family income). The same was observed according to strata of smelter job characteristics (years of work in the smelter, work in the production or administrative sector, work accidents, and sick leave) (data not shown). 

However, SF-36 mean scores showed marked differences, according to some aspects of former lead workers’ occupational history. Seventy-seven (41.4%) out of the 186 former lead workers declared that they had laboratory diagnosed metal poisoning; 129 (69.4%) claimed that their past work at the smelter hampered them from getting another job; and 156 (83.9%) claimed they were unable to get another job after dismissal. Individuals in these strata presented systematically lower health-related quality of life mean scores of health-related quality of life (Table 3).

## 4. Discussion

The most striking result of this study was the very low scores for all dimensions of health-related quality of life presented by smelter former lead workers. Their scores were far below those presented by healthy or diseased Brazilian populations. The Physical Component Summary was comparatively much lower than the Mental Component: (32.2 ± 8.6 and 40.5 ± 12.0, respectively). In a population-based random sample of 2318 Brazilian individuals, aged ≥65 years, of both sexes, mean PCS was 41.8 and MCS was 50.1. Among the 1739 individuals classified in the occupational group “qualified manual workers”, PCS and MCS were 51.5 and 51.6, respectively [27].

The low scores in general health (GH), physical functioning (PF), role limitations due to physical problems (RP), and bodily pain (BP) are statistically associated with past occupational lead exposure, probably reflecting the late effects of lead exposure on the physical health of these former workers. 

RE mean score (role limitations due to emotional problems) was particularly low (33.7 ± 12.4) compared to the other domains that most contribute to the Mental Component Summary. Part of the emotional problems referred by these former lead workers are probably related to the stigmatizing effects of their past job in the lead smelter. The health-related quality of life scores were systematically lower when the workers claimed they had a laboratory diagnosis of metal poisoning and difficulty getting another job after dismissal by the smelter (Table 3).

Patients reported outcomes, such as those measured by SF-36 scores, must be carefully analyzed. Occasionally found statistical associations between health-related quality of life scores and specific toxicological markers of lead poisoning must consider the different natures of these sets of variables. Furthermore, the cross-sectional design of this study is unable to provide evidence for causal associations. It is difficult to evaluate the role of a possible response bias. Former lead workers may have reported worse self-evaluations of their health-related quality of life because they felt unwell, worried about past occupational exposures, or because they felt stigmatized due to their difficulty returning to the job market.

**Table 2:** SF-36 dimensions raw scores (mean) in Brazilian populations and in former lead workers from the Santo Amaro da Purificação lead smelter, Brazil, 2008.

Population Characteristics	Reference	N	PF	RP	BP	GH	VT	SF	RE	MH
Healthy individuals, aged >60 years, random sample of populations from São Paulo State, Brazil	[19]	274	83.1	92.8	87.7	81.3	77.1	93.3	93.4	79.6
Individuals with stroke sequelae, aged >60 years, population random samples from São Paulo State, Brazil	[19]	93	49.0	56.1	64.8	54.9	55.3	77.9	68.1	58.4
Retired people, 55% by disablement, mean age 57 years, Belo Horizonte, Brazil	[20]	87	61.1	49.2	54.8	59.0	61.0	70.0	53.4	68.4
Patients with chronic renal failure, mean time in hemodialysis: 30 months, mean age 46 years	[21]	184	61.0	52.0	67.0	60.0	58.0	69.0	60.0	62.0
Patients with post-polio syndrome, mean age 45 years, 6 males and 9 females	[22]	15	64.7	51.7	58.0	56.4	40.0	44.2	64.1	54.9
Patients with femoral neck fracture treated by partial hip arthroplasty, mean age 83 years	[23]	30	31.7	79.2	96.9	77.6	66.2	93.8	84.4	77.2
Patients with femoral neck fracture, recently hospitalized, mean age 74 years	[24]	24	58.8	49.8	51.0	65.5	51.0	65.9	73.5	57.1
Patients with proximal femoral fracture, six months after surgical treatment, all males, mean age 74 years	[25]	16	45.9	29.7	58.6	56.9	58.4	76.6	31.3	53.0
Patients with chronic obstructive pulmonary disease, mean age 63.1 years, low income	[26]	33	30.3	24.2	68.1	63.7	57.6	55.2	66.5	64.7
Patients with chronic obstructive pulmonary disease, at long-term oxygen therapy for 2.5 ± 2.0 years, mean age 63.5 years	[26]	36	16.9	9.7	56.9	54.4	45.5	29.1	51.7	57.9
Former lead workers, Santo Amaro da Purificação, Brazil	This study	186	37.5	18.7	38.8	27.4	40.0	56.9	31.4	55.3

**Table 3:** SF-36 dimensions normalized scores (mean ± SD) in 186 former lead workers from Santo Amaro da Purificação lead smelter according to occupational history aspects, Brazil, 2008.

SF-36	Laboratory Diagnosed Metal Poisoning		*p*	Past Work in the Smelter has Hampered From Getting Another Job		*p*	Could not Get Another Job, After Dismissal by the Smelter		*p*
	Yes (*n* = 77)	No (*n* = 109)		Yes (*n* = 129)	No (*n* = 57)		Yes (*n* = 156)	No (*n* = 30)	
PF	28.2 ± 8.7	32.9 ± 11.2	0.002	28.8 ± 9.1	35.6 ± 11.9	0.001	30.4 ± 10.2	33.8 ± 11.9	0.103
RP	31.5 ± 7.3	34.4 ± 8.5	0.013	31.8 ± 7.2	36.4 ± 9.2	0.001	32.8 ± 7.5	35.7 ± 10.4	0.145
BP	34.6 ± 7.5	38.0 ± 10.1	0.010	34.3 ± 7.2	41.7 ± 11.3	0.001	36.1 ± 8.8	39.2 ± 11.5	0.166
GH	27.8 ± 6.8	31.5 ± 10.9	0.006	27.8 ± 8.0	34.9 ± 11.1	0.001	28.9 ± 9.0	35.7 ± 10.5	0.002
VT	39.9 ± 8.7	43.4 ± 11.7	0.018	39.4 ± 9.6	47.9 ± 10.9	0.001	40.9 ± 10.3	47.7 ± 11.3	0.001
SF	35.9 ± 10.9	34.5 ± 40.1	0.018	36.3 ± 11.8	43.2 ± 12.9	0.001	38.1 ± 12.2	40.1 ±1 4.1	0.410
RE	32.6 ± 12.2	34.4 ± 12.5	0.348	31.8 ± 11.4	37.8 ± 13.7	0.005	32.9 ± 11.8	37.4 ± 14.7	0.121
MH	36.0 ± 11.4	40.6 ± 13.0	0.014	35.4 ± 14.0	46.1 ± 11.5	0.001	37.6 ± 11.9	44.7 ± 14.5	0.004
PCS	30.1 ± 7.5	33.8 ± 9.0	0.003	30.6 ± 7.1	35.9 ± 10.3	0.001	31.8 ± 8.2	34.7 ± 9.9	0.087
MCS	38.6 ± 10.5	41.8 ± 12.9	0.069	37.8 ± 11.1	46.5 ± 11.8	0.001	39.5 ± 11.5	45.5 ± 13.6	0.011

## 5. Conclusions

Former lead workers presented poor health-related quality of life compared to other population-based and nosological groups from Brazil. Former lead workers who referred metal poisoning; difficulty getting another job; and those who could not get another job after smelter closure had poorer health-related quality of life. Former lead workers with poor health-related quality of life make part of the huge occupational liability left by the Santo Amaro lead smelter. 
